# The Awareness and Experience About Endoscopic Adverse Events Among South Korean Endoscopists

**DOI:** 10.5152/tjg.2023.22256

**Published:** 2023-09-01

**Authors:** Eun Hye Oh, Junseok Park, Jun Kyu Lee, Jeong Eun Shin, Sangjin Cho, Seung Bae Yoon, Young Sin Cho, Yong Hwan Kwon, Ki Bae Kim, Wan Jung Kim, Chang Nyol Paik, Hyun Chul Lim, Yoon Suk Lee, Jae-Young Jang, Tae Hee Lee, The Quality Management Committee of the Korean Society of Gastrointestinal Endoscopy (KSGE)

**Affiliations:** 1 Department of Internal Medicine, Hanyang University Guri Hospital, Hanyang Unversity, Guri, Republic of Korea; 2 Department of Internal Medicine, Digestive Disease Center, Institute for Digestive Research, Soonchunhyang University College of Medicine, Seoul, Republic of Korea; 3 Department of Internal Medicine, Dongguk University Ilsan Hospital, Gyeonggi-do, Republic of Korea; 4 Department of Internal Medicine, Dankook University College of Medicine, Cheonan, Republic of Korea; 5 Department of Internal Medicine, Seoul Good Morning Internal Medicine Clinic, Seoul, Republic of Korea; 6 Department of Internal Medicine, Eunpyoeng St. Mary’s Hospital, The Catholic University of Korea College of Medicine, Seoul, Republic of Korea; 7 Department of Internal Medicine, Soonchunhyang University College of Medicine, Cheonan, Republic of Korea; 8 Department of Internal Medicine, Kyungpook National University School of Medicine, Daegu, Republic of Korea; 9 Department of Internal Medicine, Chungbuk National University Hospital, Cheongju, Republic of Korea; 10 Department of Internal Medicine, Daehang Hospital, Seoul, Republic of Korea; 11 Department of Internal Medicine, St. Vincent’s Hospital, The Catholic University of Korea College of Medicine, Suwon, Republic of Korea; 12 Department of Internal Medicine, Yongin Severance Hospital, Yonsei University College of Medicine, Yongin, Republic of Korea; 13 Department of Internal Medicine, Inje University Ilsan Paik Hospital, Goyang, Republic of Korea; 14 Department of Internal Medicine, Kyung Hee University College of Medicine, Seoul, Republic of Korea

**Keywords:** Questionnaire, endoscopy, adverse events, endoscopist

## Abstract

**Background/Aims:**

The number of endoscopic procedures and related adverse events is increasing. We investigated South Korean endoscopists’ awareness and experience of endoscopic adverse events.

**Materials and Methods:**

We used Google Forms to conduct an online questionnaire survey among South Korean endoscopists from December 11 to 29, 2020. The survey comprised 30 questions developed by members of the Quality Management Committee of the Korean Society of Gastrointestinal Endoscopy.

**Results:**

In total, 475 endoscopists participated in the survey. Of these, 454 (95.6%) were board-certified gastroenterologists and 255 (53.7%) had >10 years of endoscopy experience. Most participants had experienced serious adverse events requiring hospitalization (80.4%, 382/475); however, only 100 (21.1%) were aware of programs for the prevention and management of adverse endoscopic events in their affiliated endoscopy centers. Most participants (98.5%, 468/475) agreed with the need for education on medical accidents for healthcare workers. Responses were inconsistent regarding the definition of adverse events formulated by the 2010 American Society for Gastrointestinal Endoscopy Workshop. Most participants were not aware of the minimal standard terminology (76.6%, 364/475) and had not used it when writing endoscopy reports (88.8%, 422/475). Responses were inconsistent regarding which events to record in endoscopy records.

**Conclusion:**

Further discussion on the nationwide adverse-event reporting system and education program for adverse events related to endoscopy is needed to ensure the safety of patients and endoscopists.

**Main Points**

- Most Korean endoscopists have experienced endoscopic adverse events. However, there are no consensus definitions of endoscopic adverse events and unified reporting system or program for their prevention and management.
- Most participants recognized the need for education on adverse endoscopic events and related medical accidents.
- This study emphasized the need for the establishment of consensus definitions and a unified reporting system and program for the prevention and management of endoscopy-related adverse events to ensure the safety of patients and endoscopists.

## Introduction

As interest in health increases, the number of screening endoscopies also rises rapidly.^1,2^ Moreover, ever greater numbers of aged people with comorbidities are undergoing screening or diagnostic endoscopy in the aging society.^3,4^ Because of the preference for minimally invasive treatments and advances in endoscopic technology, new advanced techniques in therapeutic endoscopy have been developed to replace surgery. Endoscopic resection of tumors, endoscopic stricture dilatation, and endoscopic ultrasound-guided drainage are some examples of those new advanced techniques.^5^ Endoscopic procedures, especially if performed on elderly people with multiple comorbidities, could be associated with a higher risk of adverse events. Major adverse events include bleeding, perforation, and cardiopulmonary events. Moreover, the risk increases if therapeutic interventions are conducted during endoscopic procedures.^3,6^ Therefore, it is essential for endoscopists to be aware of the risk factors for endoscopy-related adverse events, recognize their occurrence quickly, and take timely and appropriate measures.^7^

No consensus definitions of endoscopic adverse events have been established in South Korea until now. Some South Korean endoscopists confuse complications with incidents or adverse events. The lack of a standardized definition hampers the comparison of data from different hospitals and studies on quality improvement. Therefore, we used an online questionnaire survey to investigate South Korean endoscopists’ awareness and experience of endoscopic adverse events.

## Materials and Methods

The study protocol was approved by the Hanyang Universigy Guri Hospital (approval no: GURI 2022-03-014-005).

### Data Resources

A survey commissioned by the Quality Management Committee of the Korean Society of Gastrointestinal Endoscopy comprising 30 questions formulated by members of the committee (Supplementary Material 1) was conducted online using Google Forms in December 2020. The survey, targeting South Korean endoscopists who perform endoscopic procedures in South Korea, covered the participants’ baseline characteristics, the experience of endoscopic adverse events, awareness of the definition of an endoscopic adverse event, the terminology used in clinical practice, and recording of endoscopic adverse events in endoscopy reports. The survey was conducted anonymously, and all responses were collected and analyzed.

### Grouping of Participants

For comparison, the participants were divided into gastroenterologist/expert, gastroenterologist/non-expert, and non-gastroenterologist groups. The gastroenterologist/expert group (group 1) comprised endoscopists who majored in gastroenterology and had >10 years of experience in endoscopic procedures. The gastroenterologist/non-expert group (group 2) comprised endoscopists who majored in gastroenterology and had <5 years of experience in endoscopic procedures. The non-gastroenterologist group (group 3) comprised endoscopists who majored in medical fields other than gastroenterology, with no restriction on years of experience. Participants who did not satisfy any of the 3 criteria were excluded from the intergroup analysis.

### Statistical Analysis

The response rates for each question were expressed as numbers with percentages. Values were compared among groups by analysis of variance. Statistical analysis was performed using International Business Machines Statistical Package for the Social Sciences Statistics for Windows, version 25.0 (IBM Corp.; Armonk, NY, USA). Statistical significance was set at *P < *.05. 

## Results

### Baseline Characteristics of the Participants

A total of 8644 members of the Korean Society of Gastrointestinal Endoscopy were circulated by e-mail, and 475 (5.5%) members responded. Seventy-nine (16.6%), 107 (22.5%), and 21 (4.4%) participants were classified in the groups 1, 2, and 3, respectively (Table 1). The remaining 268 participants (56.4%) were excluded from the intergroup analysis.

Three hundred fifty-seven (75.2%) participants were male (*P* =.008). Approximately one-third (33.1%, 157/475) of the participants were younger than 40 years, and the age distribution of the participants differed significantly among the groups (*P* < .001). Almost all participants (95.6%, 454/475) had majored in gastroenterology (*P* < .001). The participants were mostly affiliated with clinics (24.0%, 114/475), general hospitals (28.6%, 136/475), or certified tertiary hospital/university hospitals (31.6%, 150/475). All participants in group 1 were affiliated with a certified tertiary hospital/university hospital (*P* < .001). More than half (53.7%, 255/475) of the participants had >10 years of experience in performing endoscopy. While the years of endoscopy experience differed significantly among the groups (*P* < .001), more than 80% (80.9%, 17/21) of the participants in group 3 had >10 years of experience (Table 1).

During the previous year, 61.5% (292/475) of the participants had performed >30 esophagogastroscopies and 24.8% (118/475) had performed >70 esophagogastroscopies per week. In group 2, 68.2% (73/107) of the participants had performed >30 esophagogastroscopies and 30.8% (33/107) had performed >70 esophagogastroscopies per week. In group 3, 23.8% (5/21) had performed >70 esophagogastroscopies per week (*P* < .001) (Table 1).

During the previous year, 75.3% (353/475) of the participants had performed >10 colonoscopies and 20.2% (96/475) had performed >30 colonoscopies per week. In group 2, 79.4% (85/107) had performed >10 colonoscopies and 21.5% (23/107) had performed >30 colonoscopies per week. In the group 3, 43.0% (9/21) of the participants had performed >10 colonoscopies per week (*P* = .137) (Table 1).

### Experience with Endoscopic Adverse Events

Although the rates differed significantly among the groups (*P *< .001), most of the participants (80.4%, 382/475) responded that they had experienced serious endoscopic adverse events requiring hospitalization. Almost all (96.2%, 76/79) participants in group 1 and approximately three-quarters (76.2%, 16/21) of those in group 3 had experienced serious endoscopic adverse events requiring hospitalization; fewer participants (62.6%, 67/107) in group 2 had experienced such events (Table 2).

While multiple responses were allowed, the majority (96.3%, 648/673) of the participants recognized the occurrence of an endoscopic adverse event based on the patient’s actions (60.7% [408/673], 33.6% [226/673], and 2.1% [14/673] of the responses were outpatient-clinic visits, telephone contacts, and emergency room visits, respectively). In group 1, 4 participants (3.4%, 4/673) responded that they obtained information about adverse events via the dispute mediation committee of their institutions (*P* = .002) (Table 2).

While multiple responses were allowed, most (90.3%, 485/537) participants tended to resolve endoscopic adverse events via the physician–patient relationship (288/537, 53.6%) or settlement between the medical institution and the patient (197/537, 36.7%). The rates of responses indicating that medical accidents were resolved legally were higher in group 1 (8.9%, 11/123) and group 3 (11.2%, 2/18) than in group 2 (2.3%, 2/88) (*P* = .002) (Table 2).

Approximately, one-fifth (21.1%, 100/475) of the participants were aware of programs for the prevention and management of endoscopic adverse events in their affiliated endoscopy centers (*P* = .004). More participants were aware of such programs in group 1 (27.8%, 22/79) than in group 2 (20.6%, 22/107) and group 3 (23.8%, 5/21). Moreover, less than one-fifth (18.5%, 88/475) of the participants stated that they were collecting and statistically analyzing data on endoscopic adverse events in the affiliated endoscopy centers (*P* < .001). More participants were collecting and analyzing data in group 1 (38.0%, 30/79) than in group 2 (16.8%, 18/107) and group 3 (4.8%, 1/21) (Table 2).

Most of the participants (87.8%, 417/475) stated that their medical care was influenced by the news about the imprisonment of physicians involved in endoscopic adverse events (*P* = .049). The proportion of participants affirming a negative influence of the news was higher in group 2 (92.5%, 99/107) than in group 1 (83.5%, 66/79) and group 3 (81.4%, 17/21). Almost all participants (98.5%, 468/475) agreed that there is a need for education on medical accidents for healthcare workers (*P =* .375). Also, approximately four-fifths (81.1%, 385/475) of the participants agreed with the need for a database for post-registration of delayed endoscopic adverse events (*P =* .204) (Table 2).

### Awareness of the Definition of Endoscopic Adverse Events

Awareness of the definition of endoscopic adverse events was assessed based on 8 questions regarding the statement by the American Society for Gastrointestinal Endoscopy (ASGE) workshop 2010^8^ and the Minimal Standard Terminology (MST)^9^ (Table 3).

Four hundred nine participants (86.1%, 409/475) agreed with the 2010 ASGE workshop’s proposed definition of an adverse event as one preventing completion of the planned procedure and/or resulting in hospitalization, a prolonged hospital stay, another procedure (requiring sedation/anesthesia), or subsequent medical consultation (*P* = .062). Three-quarters of the participants (75.0%, 356/475) agreed with the 2010 ASGE workshop’s suggestion that the severity of an adverse event should be graded by the degree of its disturbance to the patient and any resulting changes to the plan of care (*P* = 0.986). However, fewer participants (62.9%, 299/475) agreed that temporary ventilation support by bagging or the nasal airway during conscious sedation does not constitute an adverse event (*P* = .111). Moreover, 60.0% (285/475) of the participants stated that an unintended episode that occurred during endoscopy but resolved without interruption to the intended procedure was not an adverse event (*P* = .219) (Table 3).

Less than one-quarter (23.4%, 111/475) of the participants were aware of the MST (*P *= .009). Only 11.2% (53/475) of the participants stated that they used the MST when writing endoscopy reports (*P* = .037). Few of the participants (3.8%, 18/475) answered that they recorded adverse events in endoscopy reports in accordance with the MST (*P* = .599). Approximately two-thirds of the participants (65.9%, 313/475) agreed with the MST that the need for hospitalization, prolongation of a planned hospital stay, or an additional procedure was important in defining the severity of an adverse event (*P* = .852) (Table 3).

### Terminology for Endoscopic Adverse Events in Clinical Practice

South Korean endoscopists use the Korean word “UBALJEUNG” or “HAPBYEONGJEUNG” when communicating with patients regarding endoscopic adverse events. The Korean word “UBALJEUNG” refers to an event or symptom that occurs accidentally, and “HAPBYEONGJEUNG” refers to a disease that occurs with another disease or postoperatively. Some South Korean physicians believe that “UBALJEUNG” has a strong nuance, emphasizing value-neutral unanticipated problems that arise following medical/surgical procedures, whereas “HAPBYEONGJEUNG” involves a value judgment.

In this study, 12.8% (61/475) and 40.6% (193/475) of the participants used the terms “UBALJEUNG” and “HAPBYEONGJEUNG,” respectively, when communicating with patients suffering serious bleeding/perforation related to endoscopy; 42.6% (202/475 participants) used the 2 words interchangeably (P = .084) (Table 4).

In defining adverse events related to diagnostic endoscopy, 56.3% (267/475) and 28.0% (133/475) of the participants responded that 3 and 7 days, respectively, would be appropriate standards (*P* = .506). “Late” adverse events are events that occur later than general endoscopic adverse events but with a clear causal relationship. In this study, 46.4% (220/475) of the participants answered that 1 week would be the appropriate standard. Also, 40.4% (192/475) of the participants stated that the standard should differ according to procedure type and purpose (*P* = .698) (Table 4).

### Recording Endoscopic Adverse Events in the Endoscopy Report

Regarding temporary bleeding after biopsy using forceps that required control using epinephrine spray, 38.7% (184/475) of the participants answered that they would record the event as an adverse event in the endoscopy report (*P =* .647). Also, 20.2% (96/475) of the participants responded that they would record temporary desaturation requiring oxygen supplementation with full recovery during conscious sedation as an adverse event in the endoscopy report (*P =* .668) (Table 5).

Less than one-half (48.2%, 229/475) of the participants agreed that no record is needed in the endoscopy report if a temporary unintended episode occurs but fully resolves and the intended procedure is completed with only a temporary halt, oxygen supplementation, or administration of an antispasmodic, analgesic, or antihypertensive medication (*P =* .026). More than half (59.4%, 282/475) of the participants answered that recording of such events in the endoscopy centers’ record files is necessary, while recording in the endoscopy report is not (*P* = .526) (Table 5).

## Discussion

It is essential for endoscopists to be aware of endoscopic adverse events, including their prevention, early detection, and management. Therefore, we used an online questionnaire survey to investigate South Korean endoscopists’ awareness and experience of endoscopic adverse events. The Quality Management Committee of the Korean Society of Gastrointestinal Endoscopy developed a 30-question survey, and 475 endoscopists participated in it. Most of the participants had majored in gastroenterology; however, the endoscopists were not acquainted with the unified definition of endoscopic adverse events provided by the 2010 ASGE workshop and were not familiar with the MST. Although most of the participants had experienced serious endoscopic adverse events requiring hospitalization, only 21.1% were aware of programs for the prevention and management of endoscopic adverse events in their affiliated endoscopy centers. Most of the participants stated that their active medical care was affected by the news of the imprisonment of physicians involved in endoscopic adverse events, and almost all participants agreed with the need for healthcare workers to receive education on medical accidents. 

In addition to years of experience, other factors, such as patient- or procedure-risk factors, contribute to adverse events.^3,6^ In this study, all participants in group 1 had >10 years of experience in performing endoscopy and were affiliated with certified tertiary/university hospitals. Thus, they would have performed more high-risk endoscopic procedures in patients with risk factors. This explains why participants in group 1 had more experience with endoscopic adverse events compared with the other groups. 

Fatigue and excessive workloads result in carelessness and increase the incidence of accidents,^10,11^, which also applies to patient care in hospitals.^12^ Performing too many endoscopies per hour causes fatigue and reduced attention, both by endoscopists and other healthcare workers, and reduces attention to patient safety and the allowance of sufficient observation during endoscopy to detect significant lesions. In South Korea, starting at the age of 40 years, people are provided free biannual esophagogastroduodenoscopy for gastric cancer screening by the National Cancer Screening Program. Likewise, for colorectal cancer screening, starting at the age of 50 years, a free colonoscopy is provided in the event of the annual fecal occult blood test being positive.^13,14^ Moreover, medical fees in South Korea are very low. As a result, endoscopists typically perform large numbers of endoscopies in a short time. The percentages of participants who had performed large numbers of endoscopies in a week were higher in groups 2 and 3 than those in group 1. Large numbers of endoscopies performed within a short period by non-expert endoscopists led to an increased rate of endoscopic adverse events.^15^

Nevertheless, the percentages of the participants who were aware of programs for the prevention and management of endoscopic adverse events in their affiliated endoscopy centers and collecting and statistically analyzing data on endoscopic adverse events in their affiliated endoscopy centers were low and much lower in group 2 and group 3 than that in group 1. These data show that participants in group 2 and group 3 were not well acquainted with endoscopic adverse events and were not conducting quality improvement programs in their affiliated endoscopy centers, which are essential to prevent such events.^16^ This might be partly explained by a lack of interest related to a low level of experience of endoscopic adverse events in these groups and may reflect insufficient education on adverse events. Therefore, education on preventing, recognizing, and appropriately managing adverse events should be provided not only to endoscopists who majored in gastroenterology but also to those who did not.^17^

The proportion of participants who stated that their medical care was affected by the news of the imprisonment of physicians involved in endoscopic adverse events was higher in group 2. A lack of knowledge on preventing, recognizing, and appropriately managing adverse events might have contributed to the non-experts’ fear of medical accidents or adverse events. Although most medical accidents or adverse events are resolved via the physician–patient relationship or a settlement between the affiliated medical institution and the patient, the number of lawsuits related to endoscopic adverse events is increasing rapidly. Therefore, education should include the non-medical aspects of adverse events, such as the principles and skills of the physician–patient relationship. Indeed, almost all participants agreed with the need for healthcare workers to receive education on medical accidents.^18,19^ Endoscopists should be trained in endoscopy-related medical accidents via a minimal curriculum.^20^ Moreover, arbitration agencies, such as the courts, the South Korean Medical Dispute Mediation and Arbitration Agency, and the South Korean Consumer Agency, should render fair judgments.^21^

It is desirable that adverse events are recognized during endoscopic procedures and action is taken immediately. However, symptoms/signs of adverse events often manifest after the patient has returned home; such late events include delayed perforation^22^ and delayed bleeding.^23^ In this study, the participants learned of the adverse event most frequently as a result of the patient’s actions. To ensure favorable outcomes, it is important to take appropriate measures without delay, and patients should be informed of the symptoms/signs indicative of endoscopic adverse events and response guidelines before they return home. For example, the possibility of delayed bleeding or perforation should be described to patients undergoing endoscopic polypectomy. It would be desirable to track and check the patients’ symptoms/signs after they have returned home, but this is not easily feasible. 

In this study, most of the participants agreed with the need for a database allowing post-registration of delayed endoscopic adverse events. The registration system would be useful for collecting and analyzing data on adverse events irrespective of the time of occurrence or medical institution, thereby helping to improve overall safety related to endoscopy. However, to enable the system, definitions and terminologies for adverse events must be defined first. In this study, the responses to questions using the definitions of adverse events provided by the ASGE workshop 2010^8^ and MST^9^ showed marked variation. Also, the terminology for adverse events is not unified, and it is unclear which events need to be recorded. Thus, there is a need for endoscopists to be sufficiently educated. An atmosphere should be established in which honest and exact record-keeping does not increase the fear of the consequences of legal disputes. There should be no fear of the fact that an adverse event has occurred or of discussing it with patients and co-workers. To this end, objective and reasonable arbitration between judicial and arbitration agencies is essential.^21^ Endoscopists should be protected from legal jeopardy resulting from unavoidable adverse events.^24^

This study had the following limitations. First, not all endoscopists in South Korea participated in this study. Most participants were endoscopists working at hospital-level institutions. Considering that more than half of all endoscopies are conducted at clinic-level institutions, the participants might not have been representative of all South Korean endoscopists. Nevertheless, our results show that participants’ level of knowledge was low despite their experience of endoscopy and related adverse events. Another limitation is the low response rate of the survey. We think the response rate was low because the survey encouraged voluntary participation without forcing or granting the benefit of the response. Third, the participants were grouped according to their major and endoscopy experience. However, this was necessary to compare levels of awareness and knowledge of endoscopic adverse events among the groups. Third, we were unable to propose a unified definition of adverse events or classification of their severity and timing. More research and discussion are needed to establish a unified definition and classification. 

We evaluated South Korean endoscopists’ awareness and experience of endoscopic adverse events with the aim of improving the safety of endoscopy. We suggest that there is a need for further discussion of adverse event reporting and education. Ultimately, a nationwide system is needed that allows follow-up after endoscopy even if the patient does not re-visit the previous hospital. Thereby, developments in endoscopic techniques and minimally invasive treatment modalities will ensure the safety of patients and endoscopists. 

## Figures and Tables

**Table 1.** Baseline Characteristics of the Survey Participants

	Total (n = 475)	Comparison Among Groups			
		Group 1 (n = 79)	Group 2 (n = 107)	Group 3 (n = 21)	*P*
What is your sex?					
Male	357 (75.2%)	58 (73.4%)	69 (64.5%)	14 (66.7%)	**.008**
What is your age?					
Under 40 years	157 (33.1%)	2 (2.5%)	95 (88.8%)	2 (9.5%)	**<.001**
40s	214 (45.0%)	45 (57.0%)	12 (11.2%)	7 (33.3%)	
50s	92 (19.4%)	27 (34.2%)	0 (0.0%)	10 (47.7%)	
Over 60 years	12 (2.5%)	5 (6.3%)	0 (0.0%)	2 (9.5%)	
What is your major?					
Gastroenterology	454 (95.6%)	79 (100.0%)	107 (100.0%)	0 (0.0%)	**<.001**
Internal medicine, other than gastroenterology	8 (1.7%)	0 (0.0%)	0 (0.0%)	8 (38.1%)	
General surgery	6 (1.3%)	0 (0.0%)	0 (0.0%)	6 (28.6%)	
Pediatrics	3 (0.6%)	0 (0.0%)	0 (0.0%)	3 (14.3%)	
Family medicine	4 (0.8%)	0 (0.0%)	0 (0.0%)	4 (19.0%)	
What is your affiliated medical institution’s classification?					
Health examination center	22 (4.6%)	0 (0.0%)	10 (9.3%)	1 (4.8%)	**<.001**
Clinic	114 (24.0%)	0 (0.0%)	15 (14.0%)	7 (33.4%)	
Hospital	53 (11.2%)	0 (0.0%)	19 (17.8%)	5 (23.8%)	
General hospital	136 (28.6%)	0 (0.0%)	25 (23.4%)	4 (19.0%)	
Certified tertiary hospital/university hospital	150 (31.6%)	79 (100.0%)	38 (35.5%)	4 (19.0%)	
How many years have you performed endoscopy?					
Less than 2 years	40 (8.4%)	0 (0.0%)	40 (37.4%)	0 (0.0%)	**<.001**
More than 2 years but less than 5 years	68 (14.3%)	0 (0.0%)	67 (62.6%)	1 (4.8%)	
More than 5 years but less than 10 years	112 (23.6%)	0 (0.0%)	0 (0.0%)	3 (14.3%)	
More than 10 years but less than 15 years	100 (21.1%)	27 (34.2%)	0 (0.0%)	5 (23.8%)	
More than 15 years	155 (32.6%)	52 (65.8%)	0 (0.0%)	12 (57.1%)	
How many esophagogastroduodenoscopies do you perform per week? (Average in the past year)					
Less than 10	42 (8.8%)	11 (13.9%)	5 (4.7%)	8 (38.1%)	**<.001**
More than 10 but less than 30	141 (29.8%)	31 (39.2%)	29 (27.1%)	4 (19.0%)	
More than 30 but less than 50	116 (24.4%)	18 (22.8%)	24 (22.4%)	1 (4.8%)	
More than 50 but less than 70	58 (12.2%)	4 (5.1%)	16 (15.0%)	3 (14.3%)	
More than 70	118 (24.8%)	15 (19.0%)	33 (30.8%)	5 (23.8%)	
How many colonoscopies do you perform per week? (Average in the past year)					
Less than 10	122 (25.7%)	24 (30.4%)	22 (20.6%)	12 (57.0%)	.137
More than 10 but less than 30	257 (54.1%)	37 (46.8%)	62 (58.0%)	6 (28.6%)	
More than 30 but less than 50	52 (10.9%)	9 (11.4%)	15 (14.0%)	1 (4.8%)	
More than 50 but less than 70	19 (4.0%)	3 (3.8%)	4 (3.7%)	1 (4.8%)	
More than 70	25 (5.3%)	6 (7.6%)	4 (3.7%)	1 (4.8%)	

Group 1: Gastroenterologist/expert group comprised endoscopists who majored in gastroenterology and had >10 years of experience in endoscopic procedures; Group 2: gastroenterologist/non-expert group comprised endoscopists who majored in gastroenterology and had <5 years of experience in endoscopic procedures; Group 3: non-gastroenterologist group comprised endoscopists who majored in medical fields other than gastroenterology, with no restriction on years of experience; participants who did not satisfy any of the 3 conditions defining the groups were excluded from the intergroup analysis (268 participants, 56.4%).

**Table 2.** Experience of Endoscopic Adverse Events

	Total (n = 475)	Comparison Among Groups			
		Group 1 (n = 79)	Group 2 (n = 107)	Group 3 (n = 21)	*P*
Have you ever experienced a serious endoscopic adverse event requiring hospitalization?					
Yes	382 (80.4%)	76 (96.2%)	67 (62.6%)	16 (76.2%)	**<.001**
No	93 (19.6%)	3 (3.8%)	40 (37.4%)	5 (23.8%)	
How have you obtained information on endoscopic adverse events? **(**Multiple responses are allowed)*					
Noticed during or immediately after endoscopy	11 (1.6%)	3 (2.6%)	1 (0.7%)	0 (0.0%)	**.002**
Noticed during the hospitalization period (for hospitalized patients)	7 (1.0%)	2 (1.7%)	1 (0.7%)	2 (6.7%)	
Telephone contact with the patient	226 (33.6%)	30 (25.9%)	47 (32.2%)	11 (36.7%)	
Outpatient clinic visit of the patient	408 (60.7%)	70 (60.3%)	93 (63.7%)	17 (56.6%)	
Emergency room visit of the patient	14 (2.1%)	6 (5.2%)	4 (2.7%)	0 (0.0%)	
Unaware of the occurrence of an adverse event	2 (0.3%)	1 (0.9%)	0 (0.0%)	0 (0.0%)	
Via the dispute mediation committee of the affiliated medical institution	5 (0.7%)	4 (3.4%)	0 (0.0%)	0 (0.0%)	
How have you resolved medical disputes involving endoscopic adverse events? (Multiple responses are allowed)¶					
Physician–patient relationship	288 (53.6%)	59 (48.1%)	55 (62.5%)	8 (44.4%)	**.002**
Settlement between the affiliated medical institution and the patient	197 (36.7%)	42 (34.1%)	23 (26.1%)	8 (44.4%)	
The Korea Medical Association Medical Indemnity Mutual	2 (0.4%)	0 (0.0%)	0 (0.0%)	0 (0.0%)	
Mediation by the Korea Medical Dispute Mediation and Arbitration Agency or the Korean Consumer Agency	30 (5.6%)	11 (8.9%)	8 (9.1%)	0 (0.0%)	
Lawsuit	20 (3.7%)	11 (8.9%)	2 (2.3%)	2 (11.2%)	
Are there programs for the prevention and management of endoscopic adverse events in the affiliated endoscopy centers?					
Yes	100 (21.1%)	22 (27.8%)	22 (20.6%)	5 (23.8%)	**.004**
No	252 (53.0%)	40 (50.7%)	43 (40.1%)	9 (42.9%)	
Do not know	123 (25.9%)	17 (21.5%)	42 (39.3%)	7 (33.3%)	
Do you collect and statistically analyze data on endoscopic adverse events in the affiliated endoscopy centers?					
Yes	88 (18.5%)	30 (38.0%)	18 (16.8%)	1 (4.8%)	**<.001**
No	311 (65.5%)	33 (41.7%)	65 (60.8%)	18 (85.7%)	
Do not know	76 (16.0%)	16 (20.3%)	24 (22.4%)	2 (9.5%)	
Has your medical care been affected by the news of the imprisonment of physicians involved in endoscopic adverse events?					
Yes	417 (87.8%)	66 (83.5%)	99 (92.5%)	17 (81.4%)	**.049**
No	27 (5.7%)	10 (12.7%)	2 (1.9%)	2 (9.3%)	
Difficult to answer	31 (6.5%)	3 (3.8%)	6 (5.6%)	2 (9.3%)	
Do you think education on medical accidents is needed for healthcare workers?					
Yes	468 (98.5%)	79 (100.0%)	104 (97.2%)	20 (95.2%)	.375
No	1 (0.2%)	0 (0.0%)	0 (0.0%)	0 (0.0%)	
Difficult to answer	6 (1.3%)	0 (0.0%)	3 (2.8%)	1 (4.8%)	
Do you think a database for post-registration of delayed endoscopic adverse events is needed?					
Yes	385 (81.1%)	67 (84.8%)	93 (86.9%)	17 (81.0%)	.204
No	33 (6.9%)	7 (8.9%)	3 (2.8%)	2 (9.5%)	
Difficult to answer	57 (12.0%)	5 (6.3%)	11 (10.3%)	2 (9.5%)	

Group 1: gastroenterologist/expert group comprised endoscopists who majored in gastroenterology and had >10 years of experience in endoscopic procedures; Group 2: gastroenterologist/non-expert group comprised endoscopists who majored in gastroenterology and had <5 years of experience in endoscopic procedures; Group 3: non-gastroenterologist group comprised endoscopists who majored in medical fields other than gastroenterology, with no restriction on years of experience.

*Analyzed 673 answers by 475 responders; ^¶^analyzed 537 answers by 416 responders.

**Table 3.** Awareness of the Definition of an Endoscopic Adverse Event

	Total (n = 475)	Comparison Among Groups			
		Group 1 (n = 79)	Group 2 (n = 107)	Group 3 (n = 21)	*P*
The ASGE workshop 2010						In the ASGE Workshop 2010, an adverse event was defined as an event that prevents completion of a planned procedure and/or results in hospitalization, a prolonged hospital stay, another procedure (requiring sedation/anesthesia), or subsequent medical consultation. Do you agree?					
Yes	409 (86.1%)	67 (84.8%)	84 (78.5%)	17 (81.0%)	.062
No	20 (4.2%)	3 (3.8%)	7 (6.5%)	0 (0.0%)	
Difficult to answer	46 (9.7%)	9 (11.4%)	16 (15.0%)	4 (19.0%)	
The ASGE workshop 2010 graded the severity of an adverse event based on the degree of its disturbance to the patient and any resulting changes in the plan of care. Do you agree?					
Yes	356 (75.0%)	61 (77.2%)	79 (73.9%)	17 (80.9%)	.986
No	31 (6.5%)	4 (5.1%)	7 (6.5%)	1 (4.8%)	
Difficult to answer	88 (18.5%)	14 (17.7%)	21 (19.6%)	3 (14.3%)	
The ASGE workshop 2010 did not define temporary ventilation support by bagging or nasal airway use during conscious sedation to be an adverse event. Do you agree?					
Yes	299 (62.9%)	48 (60.8%)	56 (52.4%)	14 (66.7%)	.111
No	93 (19.6%)	20 (25.3%)	27 (25.2%)	4 (19.0%)	
Difficult to answer	83 (17.5%)	11 (13.9%)	24 (22.4%)	3 (14.3%)	
If an unintended episode occurred during endoscopy but was resolved without interrupting the completion of the intended procedure, would you consider it an adverse event?					
Yes	285 (60.0%)	53 (67.1%)	58 (54.2%)	15 (71.5%)	.219
No	113 (23.8%)	14 (17.7%)	26 (24.3%)	2 (9.5%)	
Difficult to answer	77 (16.2%)	12 (15.2%)	23 (21.5%)	4 (19.0%)	
MST					
Have you heard of the MST?					
Yes	111 (23.4%)	29 (36.7%)	17 (15.9%)	4 (19.0%)	**.009**
No	364 (76.6%)	50 (63.3%)	90 (84.1%)	17 (81.0%)	
Do you use the MST when writing an endoscopy report?					
Yes	53 (11.2%)	16 (20.3%)	10 (9.3%)	3 (14.3%)	**.037**
No	422 (88.8%)	63 (79.7%)	97 (90.7%)	18 (85.7%)	
Do you record adverse events in accordance with the MST in the endoscopy report?					
Yes	18 (3.8%)	5 (6.3%)	3 (2.8%)	1 (4.8%)	.599
No	457 (96.2%)	74 (93.7%)	104 (97.2%)	20 (95.2%)	
In the MST, hospitalization, prolongation of a planned hospital stay, and the need for an additional procedure for recovery are important in defining the severity of an adverse event. Do you agree?					
Yes	313 (65.9%)	51 (64.6%)	76 (71.0%)	12 (57.2%)	.852
No	29 (6.1%)	6 (7.6%)	5 (4.7%)	2 (9.5%)	
Difficult to answer	133 (28.0%)	22 (27.8%)	26 (24.3%)	7 (33.3%)	

Group 1: gastroenterologist/expert group comprised endoscopists who majored in gastroenterology and had >10 years of experience in endoscopic procedures; Group 2: gastroenterologist/non-expert group comprised endoscopists who majored in gastroenterology and had <5 years of experience of endoscopic procedures; Group 3: non-gastroenterologist group comprised endoscopists who majored in medical fields other than gastroenterology, with no restriction on years of experience.

ASGE, American Society for Gastrointestinal Endoscopy; MST, Minimal Standard Terminology.

**Table 4.** Terminology on Endoscopic Adverse Events in Clinical Practice

	Total (n = 475)	Comparison Among Groups			
		Group 1 (n = 79)	Group 2 (n = 107)	Group 3 (n = 21)	*P*
Which of the following words do you use when communicating with patients suffering serious bleeding/perforation related to endoscopy?					
UBALJEUNG	61 (12.8%)	9 (11.4%)	10 (9.3%)	5 (23.8%)	.084
HAPBYEONGJEUNG	193 (40.6%)	28 (35.4%)	57 (53.3%)	4 (19.0%)	
UBALJEUNG/HAPBYEONGJEUNG, without distinction	202 (42.6%)	40 (50.7%)	37 (34.6%)	11 (52.4%)	
Difficult to answer	19 (4.0%)	2 (2.5%)	3 (2.8%)	1 (4.8%)	
In the definition of an adverse event related to diagnostic endoscopy, how many days do you think would be an appropriate standard?					
Within 1 day	17 (3.6%)	3 (3.8%)	4 (3.7%)	0 (0.0%)	.506
Within 2 days	4 (0.8%)	0 (0.0%)	1 (0.9%)	0 (0.0%)	
Within 3 days	267 (56.3%)	49 (61.9%)	60 (56.2%)	12 (57.2%)	
Within 7 days	133 (28.0%)	18 (22.8%)	25 (23.4%)	5 (23.8%)	
Within 14 days	19 (4.0%)	4 (5.1%)	7 (6.5%)	2 (9.5%)	
Within 21 days	2 (0.4%)	0 (0.0%)	2 (1.9%)	0 (0.0%)	
The standard should differ according to the adverse event	3 (0.6%)	1 (1.3%)	0 (0.0%)	0 (0.0%)	
Difficult to answer	30 (6.3%)	4 (5.1%)	8 (7.4%)	2 (9.5%)	
“Late adverse event” is an event that occurs later than general endoscopic adverse events, but with a clear causal relationship. For a “late adverse event,” how many days do you think should be the appropriate standard?					
1 week	220 (46.4%)	39 (49.4%)	43 (40.2%)	11 (52.4%)	.698
2 weeks	49 (10.3%)	6 (7.6%)	13 (12.1%)	4 (19.0%)	
3 weeks	1 (0.2%)	0 (0.0%)	0 (0.0%)	0 (0.0%)	
4 weeks	13 (2.7%)	3 (3.8%)	1 (0.9%)	0 (0.0%)	
The standard should differ depending on the procedure type and purpose	192 (40.4%)	31 (39.2%)	50 (46.8%)	6 (28.6%)	

Group 1: gastroenterologist/expert group comprised endoscopists who majored in gastroenterology and had >10 years of experience in endoscopic procedures; Group 2: gastroenterologist/non-expert group comprised endoscopists who majored in gastroenterology and had <5 years of experience in endoscopic procedures; Group 3: non-gastroenterologist group comprised endoscopists who majored in medical fields other than gastroenterology, with no restriction on years of experience.

**Table 5.** Recording Endoscopic Adverse Events in the Endoscopy Report

	Total (n = 475)	Comparison Among Groups			
		Group 1 (n = 79)	Group 2 (n = 107)	Group 3 (n = 21)	*P*
Do you record temporary bleeding after forceps biopsy that is controlled by epinephrine spray as an adverse event in the endoscopy report?					
Yes	184 (38.7%)	29 (36.7%)	44 (41.1%)	7 (33.3%)	.647
No	275 (57.9%)	49 (62.0%)	59 (55.2%)	12 (57.2%)	
Difficult to answer	16 (3.4%)	1 (1.3%)	4 (3.7%)	2 (9.5%)	
Do you record temporary desaturation requiring oxygen supplementation with full recovery during conscious sedation as an adverse event in the endoscopy report?					
Yes	96 (20.2%)	16 (20.3%)	22 (20.6%)	3 (14.3%)	.668
No	354 (74.5%)	58 (73.4%)	83 (77.5%)	16 (76.2%)	
Difficult to answer	25 (5.3%)	5 (6.3%)	2 (1.9%)	2 (9.5%)	
Do you agree that endoscopy report recording is not necessary for a temporary unintended episode that fully resolves, and the intended procedure finishes with only a temporary halt, oxygen supplementation, or administration of an antispasmodic, analgesic, or antihypertensive medication?					
Yes (does not need to be recorded)	229 (48.2%)	37 (46.9%)	45 (42.1%)	7 (33.3%)	**.026**
No (needs to be recorded)	193 (40.6%)	34 (43.0%)	50 (46.7%)	7 (33.3%)	
Difficult to answer	53 (11.2%)	8 (10.1%)	12 (11.2%)	7 (33.3%)	
Do you agree that recording in the endoscopy centers’ records is necessary, even though recording in the endoscopy report is not necessary if a temporary unintended episode occurs but fully resolves, and the intended procedure finishes with only a temporary halt, oxygen supplementation, or administration of an antispasmodic, analgesic, or antihypertensive medication?					
Yes (needs to be recorded)	282 (59.4%)	48 (60.7%)	67 (62.6%)	13 (62.0%)	.526
No (does not need to be recorded)	113 (23.8%)	19 (24.1%)	18 (16.8%)	4 (19.0%)	
Difficult to answer	80 (16.8%)	12 (15.2%)	22 (20.6%)	4 (19.0%)	

Group 1: Gastroenterologist/expert group comprised endoscopists who majored in gastroenterology and had >10 years of experience in endoscopic procedures; Group 2: gastroenterologist/non-expert group comprised endoscopists who majored in gastroenterology and had <5 years of experience in endoscopic procedures; Group 3: non-gastroenterologist group comprised endoscopists who majored in medical fields other than gastroenterology, with no restriction on years of experience.

## Supplementary Material 1.Thirty Questions on Endoscopic Adverse Events

### Section 1. Baseline characteristics of the survey participants

**What is your sex?**
  - Male
  - Female
**What is your age?**
  - Under 40 years
  - 40s
  - 50s
  - Over 60 years
**What is your major? **
  - Gastroenterology
  - Internal medicine, other than gastroenterology
  - General surgery
  - Pediatrics
  - Family medicine
**What is your affiliated medical institution’s classification?**
  - Health examination center
  - Clinic
  - Hospital
  - General hospital
  - Certified tertiary hospital/university hospital
**How many years have you performed endoscopy?**
  - Less than 2 years
  - More than 2 years but less than 5 years
  - More than 5 years but less than 10 years
  - More than 10 years but less than 15 years
  - More than 15 years
**How many esophagogastroduodenoscopies do you perform per week? (Average in the past year)**
  - Less than 10
  - More than 10 but less than 30
  - More than 30 but less than 50
  - More than 50 but less than 70
  - More than 70
**How many colonoscopies do you perform per week? (Average in the past year)**
Less than 10
More than 10 but less than 30
More than 30 but less than 50
More than 50 but less than 70
More than 70

### Section 2. Experience of endoscopic adverse events

**Have you ever experienced a serious endoscopic adverse event requiring hospitalization?**
  - Yes
  - No
**How have you obtained information on endoscopic adverse events? (multiple responses are allowed)**
  - Noticed during or immediately after endoscopy
  - Noticed during the hospitalization period (for hospitalized patients)
  - Telephone contact with the patient
  - Outpatient clinic visit of the patient
  - Emergency room visit of the patient
  - Unaware of the occurrence of an adverse event
  - Via the dispute mediation committee of the affiliated medical institution
**How have you resolved medical disputes involving endoscopic adverse events? (Multiple responses are allowed)**
  - Physician–patient relationship
  - Settlement between the affiliated medical institution and the patient
  - The Korea Medical Association Medical Indemnity Mutual
  - Mediation by the Korea Medical Dispute Mediation and Arbitration Agency or the Korean Consumer Agency
  - Lawsuit
**Are there programs for the prevention and management of endoscopic adverse events in the affiliated endoscopy centers?**
  - Yes
  - No
  - Do not know
**Do you collect and statistically analyze data on endoscopic adverse events in the affiliated endoscopy centers?**
  - Yes
  - No
  - Do not know
**Has your medical care been affected by the news of the imprisonment of physicians involved in endoscopic adverse events?**
  - Yes
  - No
  - Difficult to answer
**Do you think education on medical accidents is needed for healthcare workers?**
  - Yes
  - No
  - Difficult to answer
**Do you think a database for post-registration of delayed endoscopic adverse events is needed?**
Yes
No
Difficult to answer

### Section 3. Awareness of the definition of an endoscopic adverse event

**[The American Society for Gastrointestinal Endoscopy (ASGE) workshop 2010]**

**In the ASGE workshop 2010, an adverse event was defined as an event that prevents completion of a planned procedure and/or results in hospitalization, a prolonged hospital stay, another procedure (requiring sedation/anesthesia), or subsequent medical consultation. Do you agree? **
  - Yes
  - No
  - Difficult to answer
**The ASGE workshop 2010 graded the severity of an adverse event based on the degree of its disturbance to the patient and any resulting changes in the plan of care. Do you agree?**
  - Yes
  - No
  - Difficult to answer
**The ASGE workshop 2010 did not define temporary ventilation support by bagging or nasal airway use during conscious sedation to be an adverse event. Do you agree?**
  - Yes
  - No
  - Difficult to answer
**|If an unintended episode occurred during endoscopy but resolved without interrupting completion of the intended procedure, would you consider it an adverse event?**
  - Yes
  - No
  - Difficult to answer

**Minimal Standard Terminology (MST)**

**Have you heard of the MST?**
  - Yes
  - No
**Do you use the MST when writing an endoscopy report?**
  - Yes
  - No
**Do you record adverse events in accordance with the MST in the endoscopy report?**
  - Yes
  - No
**In the MST, hospitalization, prolongation of a planned hospital stay, and the need for an additional procedure for recovery are important in defining the severity of an adverse event. Do you agree?**
Yes
No
Difficult to answer

### Section 4. Terminology on endoscopic adverse events in clinical practice

**Which of the following words do you use when communicating with patients suffering serious bleeding/perforation related to endoscopy?**
  - UBALJEUNG
  - HAPBYEONGJEUNG
  - UBALJEUNG/ HAPBYEONGJEUNG, without distinction
  - Difficult to answer
**In the definition of an adverse event related to diagnostic endoscopy, how many days do you think would be an appropriate standard?**
  - Within 1 day
  - Within 2 days
  - Within 3 days
  - Within 7 days
  - Within 14 days
  - Within 21 days
  - The standard should differ according to the adverse event
  - Difficult to answer
**“Late adverse event” is an event that occurs later than general endoscopic adverse events, but with a clear causal relationship. For a “late adverse event,” how many days do you think should be the appropriate standard?**
1 week
2 weeks
3 weeks
4 weeks
The standard should differ depending on the procedure type and purpose

### Section 5. Recording endoscopic adverse events in the endoscopy report

**Do you record temporary bleeding after forceps biopsy that is controlled by epinephrine spray as an adverse event in the endoscopy report?**
  - Yes
  - No
  - Difficult to answer
**Do you record temporary desaturation requiring oxygen supplementation with full recovery during conscious sedation as an adverse event in the endoscopy report?**
  - Yes
  - No
  - Difficult to answer
**Do you agree that endoscopy report recording is not necessary for a temporary unintended episode that fully resolves, and the intended procedure finishes with only a temporary halt, oxygen supplementation, or administration of an antispasmodic, analgesic, or antihypertensive medication?**
  - Yes (does not need to be recorded)
  - No (needs to be recorded)
  - Difficult to answer
**Do you agree that recording in the endoscopy centers’ records is necessary, even though recording in the endoscopy report is not necessary, if a temporary unintended episode occurs but fully resolves, and the intended procedure finishes with only a temporary halt, oxygen supplementation, or administration of an antispasmodic, analgesic, or antihypertensive medication?**
Yes (needs to be recorded)
No (does not need to be recorded)
Difficult to answer
